# Supplementary material for: Causal interplay between lactose intolerance and gut microbiota: a combined bidirectional Mendelian randomization and in vivo validation study
Source: Front Nutr. 2026 Jun 1;13:1803337. doi: 10.3389/fnut.2026.1803337 (PMC13265576; doi:10.3389/fnut.2026.1803337)
Supplement: Supplementary file 1 [file Data_Sheet_1.zip › supplementary materials/Forward/leave one out cross validation/ebi-a-GCST90027646.finngen_R12_E4_LACTONAS.pdf]

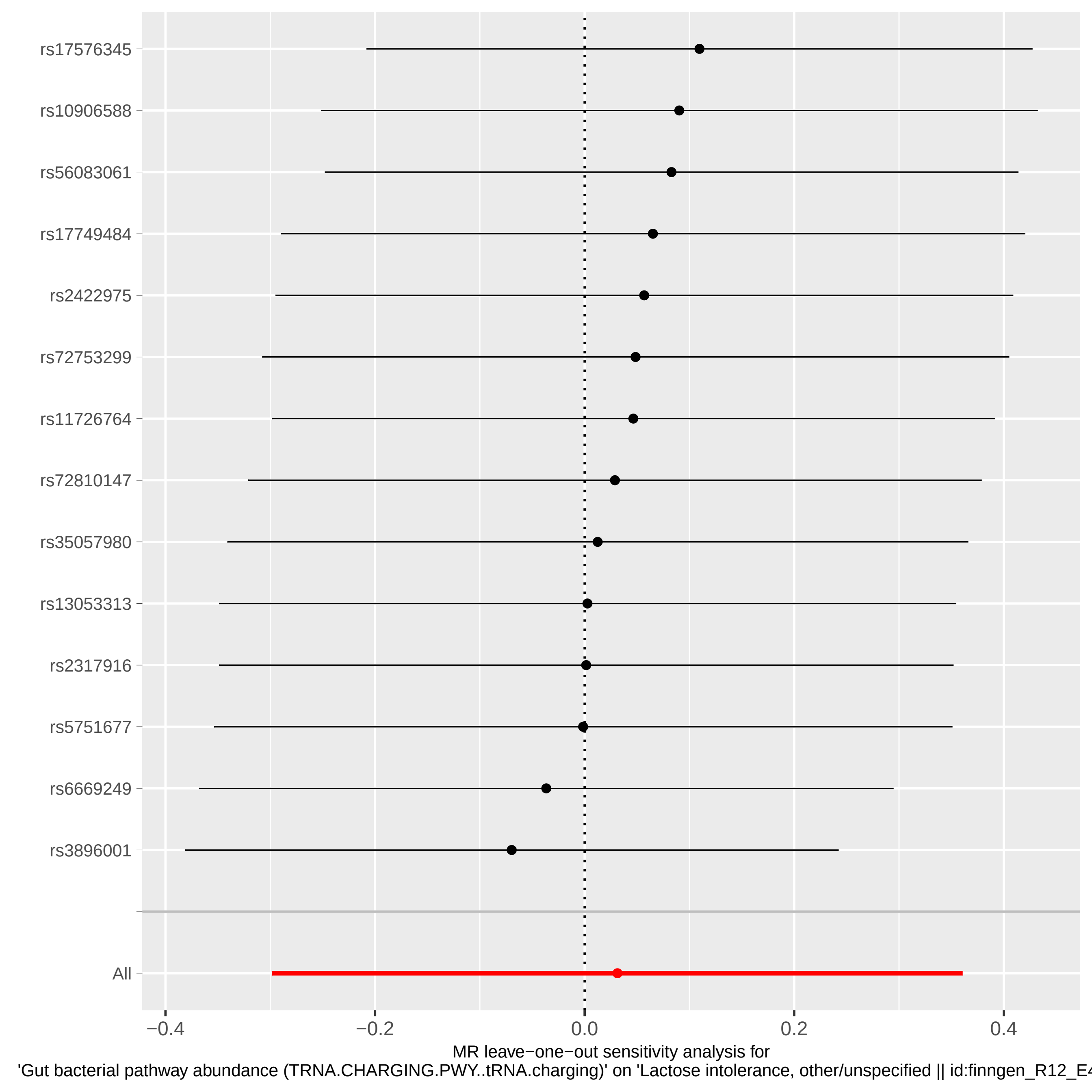
rs17576345
rs10906588
rs56083061
rs17749484
rs2422975
rs72753299
rs11726764
rs72810147
rs35057980
rs13053313
rs2317916
rs5751677
rs6669249
rs3896001
All
−0.4
−0.2
0.0
0.2
0.4
MR leave−one−out sensitivity analysis for
'Gut bacterial pathway abundance (TRNA.CHARGING.PWY..tRNA.charging)' on 'Lactose intolerance, other/unspecified || id:finngen_R12_E4
